# Ursodeoxycholic acid after common bile duct stones removal for prevention of recurrence: A systematic review and meta-analysis of randomized controlled trials

**DOI:** 10.1097/MD.0000000000013086

**Published:** 2018-11-09

**Authors:** Xun Chen, Xiao-Ru Yan, Li-Ping Zhang

**Affiliations:** a Beijing University of Chinese Medicine; b Department of Gastroenterology, Dongfang Hospital of Beijing University of Chinese Medicine; c Guang’anmen Hospital, China Academy of Chinese Medical Sciences, Beijing, China.

**Keywords:** common bile duct stones, meta-analysis, protocol, recurrence, ursodeoxycholic acid

## Abstract

**Introduction:**

The recurrence rate of common bile duct stones (CBDS) after removal has been reported to exceed 10% and no established pharmacologic treatment exists for the prevention of recurrent CBDS. Many studies indicated ursodeoxycholic acid (UDCA) has the potential to prevent the recurrence of CBDS. The aim of this systematic review is to evaluate the effects of UDCA for prevention of recurrence after common bile duct stones removal.

**Methods and analysis:**

We will systematically screen all randomized controlled trials (RCTs) published through electronically and hand searching. The following search engines including Ovid Medline, EMBASE, Cochrane CENTRAL, Proquest, Scopus, Web of Science, Pubmed, the Chinese Biomedical Literature Database, the China National Knowledge Infrastructure, VIP Information, Wanfang Data. Supplementary sources will be searched including gray literature, conference proceedings, and potential identified publications in OpenGrey.eu and Google Scholar databases. Two reviewers will independently conduct the trial inclusion, data extraction and assess the quality of studies. The recurrence rate of CBDS will be assessed as the primary outcomes. The adverse event that required discontinuation of UDCA intervention and the drop-outs (lost to follow-up) before the end of the study will be measured as secondary outcomes. Methodological quality will be evaluated according to the Cochrane risk of bias. All analyses will be applied by RevMan (version 5.3).

**Results:**

This systemic review and meta-analysis will evaluate the effects of UDCA for prevention of recurrence after CBDS removal in RCTs.

**Conclusion:**

Our study will provide evidence to judge whether UDCA is an effective intervention to prevent the recurrence after CBDS removal.

## 1 Introduction

Common bile duct stones (CBDS) is a common biliary tract disease. The past data indicates that the prevalence of CBDS among patients with symptomatic gallstones lies between 10% and 20%.^[1–6]^ CBDS is the most prevalent disease in Asia and could lead to lots of complications, such as pyogenic cholangitis and pancreatitis, which may endanger health and always occur without warning.^[7]^ In clinic, The guidelines ^[7–9]^ recommend Endoscopic Retrograde Cholangiopancreatography (ERCP) or laparoscopic surgery as the standard treatments to remove CBDS, and they usually work well.

However, the recurrence rate of CBDS has been observed in 5% to 20% according to European Association for the Study of the Liver (EASL).^[8]^ An Asian research shows that it is more than 10%.^[10]^ As a result, patients have to undergo ERCP or laparoscopic surgery again. Thus, it is required to find effective approaches to prevent the recurrence of CBDS.

Nowadays, as The Clinical Practice Guidelines ^[7–9]^ formulated by EASL said, no general recommendations could be given for the pharmacological prevention of recurrent bile duct stones, which bases on the result of a randomized controlled trial.^[11]^ This trial studied the difficult to extract CBDS, but not the prevention of the recurrence. However, many RCTs indicated that ursodeoxycholic acid (UDCA) may be a novel treatment strategy to prevent the recurrence of CBDS.^[12,13]^ So that it is essential to make a systematic review to evaluate the effect of UDCA.

UDCA, as a hydrophilic bile acid, has been used widely in clinical practice and has a definite efficacy and safety profile to treat various diseases among the liver, the biliary tract, and the digestive system. UDCA can reduce the saturation of cholesterol in bile by inhibiting the intestinal reabsorption of cholesterol and reducing the secretion of cholesterol into the bile,^[14–16]^ and improve the excretion of bile by increasing the flow rate and volume of bile, and thus it may be effective on prevention the recurrence of CBDS by promoting cholesterol stone dissolved gradually and improving cholestasis.

Although some high-quality randomized controlled clinical researches (RCTs) have been published for the past few years, there is no systematic review on UDCA for preventing the recurrence of CBDS after removal. Therefore, it is necessary to collect researches and assessment to provide up-to-date evidence for CBDS management. In this systematic review and planned meta-analysis, we will identify and evaluate the effects of UDCA for the prevention of recurrence after CBDS removal.

## 2 Methods

### 2.1 Registration

This systematic review protocol was drafted according to the Cochrane handbook for systematic reviews of interventions^[17]^ as well as the Preferred Reporting Items for Systematic Reviews and Meta-Analysis Protocols (PRISMA-P)statement.^[18]^ This protocol has been registered in the PROSPERO international prospective register of systematic reviews (registration number CRD42018098725).

### 2.2 Eligibility criteria

Studies to be incorporated into this systematic review will be selected based on the criteria specified. Our aim is to evaluate the effects of UDCA for prevention of recurrence after CBDS removal in randomized controlled trials (RCTs).

#### 2.2.1 Inclusion criteria

##### 2.2.1.1 Participants

Participants are adults aged 18 or older who had undergone a procedure for removal of CBDS. There will be no limitations on gender, education, ethnicity and removal procedure (laparoscopic surgery, Endoscopic Retrograde Cholangiopancreatography, Laparotomy and so on).

##### 2.2.1.2 Interventions

UDCA from any dosage (10 mg/kg/d, 500 mg, 750 mg and so on) and frequency (once, twice, or 3 times per day) of administration will be compared against placebo, no intervention. We will only consider UDCA postoperatively administrated.

##### 2.2.1.3 Study design

RCTs.

#### 2.2.2 Exclusion criteria

##### 2.2.2.1 Participants

Studies, where the majority of participants are post gastrectomy, cancer, or fulminant hepatitis being current treated complete obstruction of the biliary tract, alcohol abuse, pregnant, and age under 18 patients will not be considered eligible for inclusion.

##### 2.2.2.2 Interventions

Studies involving combination another bile acid formulation (e.g., Urso or Chino capsule), bile acid adsorbent (cholestimide or Questran), or cholagogue (e.g., dehydrocholic acid, Supacal, Felviten, and Inchinkoto) as interventions will not be considered eligible for inclusion.

### 2.3 Measure outcomes

We will include studies that report any of the following outcomes:

- Primary outcomes: the recurrence rate of CBDS
- Second outcomes: any adverse event that required discontinuation of UDCA intervention. Drop-outs (lost to follow up) before the end of the study.

### 2.4 Search methods for primary studies

#### 2.4.1 Electronic searches

We will search the following electronic databases without language and publication status restrictions, up to 1 October 2018: Ovid Medline, EMBASE, Cochrane CENTRAL, Proquest, Scopus, Web of Science, Pubmed, the Chinese Biomedical Literature Database, the China National Knowledge Infrastructure, VIP Information, Wanfang Data. Search terms describing choledocholithiasis, prevention and ursodeoxycholic acid (UDCA) interventions will be combined. The search strategy will be designed with the assistance of a trained librarian.

#### 2.4.2 Search strategy

We will use the following MeSH terms, with associated keywords

1. participants(choledocholithiasis, common bile duct stone, common bile duct calculi, choledocholith, CBDS)
2. interventions(ursodeoxycholic acid, UDCA, ursodiol, ursofalk)
3. outcomes(prevention, recurrence)
4. study design(Randomized controlled trial).

Additionally, we will manually search OpenGrey.eu and Google Scholar databases (http://www.clinicaltrials.gov; http://www.who.int/ictrp/en/; http://www.google.cn) to find relevant unpublished studies in grey literature to find relevant unpublished studies in grey literature and avoid the risk of missing eligible RCTs. The search strategy will be adapted for each of the aforementioned electronic databases (see Table 1 ).

**Table 1:**
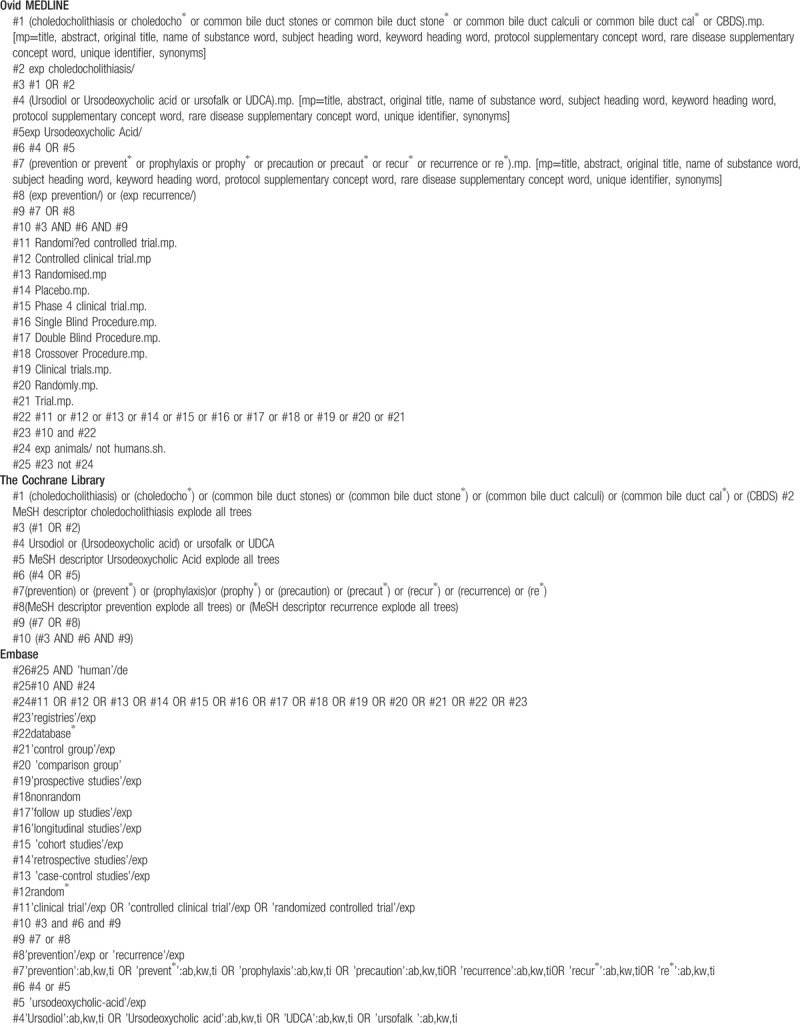
Electronic search strategies.

**Table 1 (Continued):**
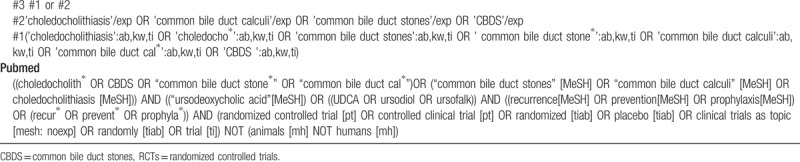
Electronic search strategies.

### 2.5 Data collection and analysis

#### 2.5.1 Selection of studies

All articles identified from the initial electronic search process will be imported into an EndNote X7 library and duplicates are removed. Two reviewers (Xun Chen and Xiao-Ru Yan) will screen references by reviewing titles and abstracts to select potentially related studies independently. The full text of the selected study will be assessed by them together to determine whether the study meets the inclusion criteria. Any disagreements in opinion will be resolved after discussion until consensus is reached. If consensus cannot be reached, the third author (Li-Ping Zhang) will arbitrate.

#### 2.5.2 Data extraction and management

**Figure 1:**
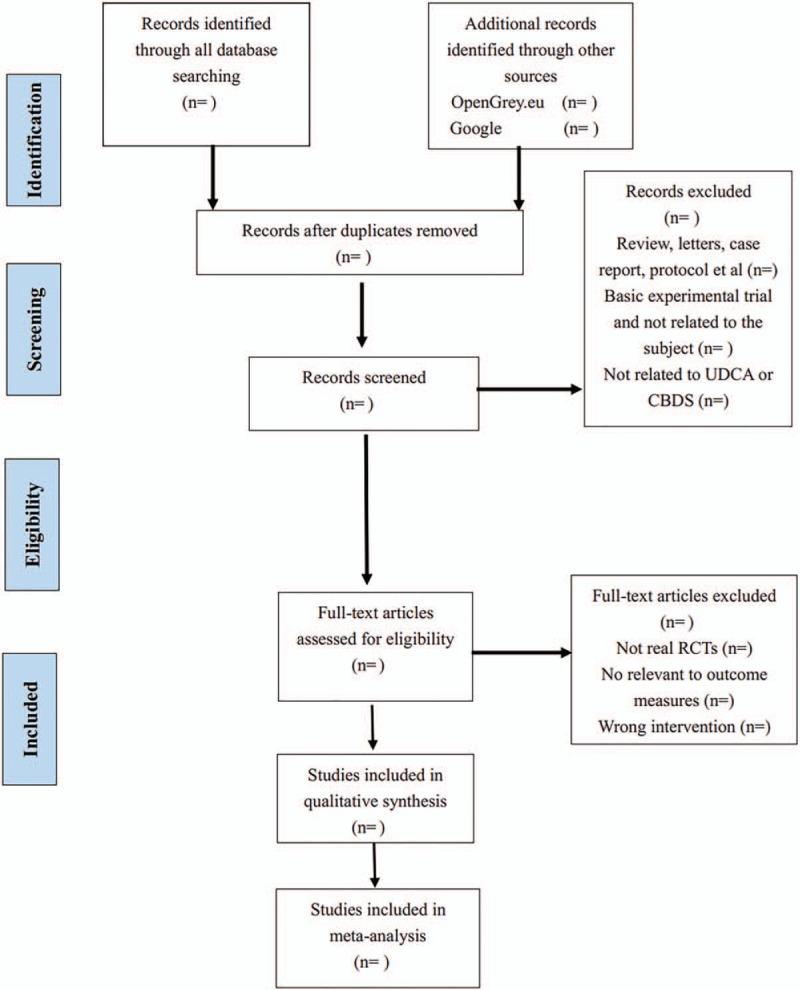
The PRISMA flow chart. PRISMA = Preferred Reporting Items for Systematic Reviews and Meta-Analysis.

All the data from studies that whether have been published or not will be described in a table. Two reviewers (Xun Chen and Xiao-Ru Yan) will extract the data of selected trials using a paper date extraction form Including: the included trials, first author, journal source, publication time, study design and key elements of quality evaluation, detail information regarding the patients, the treatment and groups, outcomes, relevant indicators of bias risk assessment, and adverse events. Any divergences will be resolved by discussion until consensus is reached. The authors of the studies will be contacted for clarification if necessary. Any remaining disagreements will be resolved after consulting a gastroenterologist or statistician. Moreover, the excluded studies after review will also be documented. All data will be input the Review Manager 5 software for meta-analysis. The details of selecting process will be presented in the PRISMA flow chart (see Fig. 1.)

### 2.6 Assessment of risk of bias in included studies

Studies that meet the criteria will be assessed for method quality/risk of bias and include the basis of method quality. We will use the following 12 criteria of Cochrane Risk of Bias Instrument to evaluated: randomization, concealed allocation, blinding, incomplete data, baseline comparability, intention-to-treat analysis, cointerventions and outcome assessment timing, and score each of the individual criteria as “high risk”, “low risk”, or “unclear risk”.^[17]^ Reviewers will not participate in risk of bias assessment for studies if they were the coauthors.

### 2.7 Measures of treatment effect

We will analyze dichotomous data through calculating the relative risks or risk ratios (RR) with 95% confidence intervals. When RR is less than 1.0, it indicates that UDCA may prevent the incidence of recurrent CBDS.

### 2.8 Dealing with missing data

Missing data or drop-outs will be evaluated. We will be contacted the authors of the studies to supply missing data and make adjustments after discussion. If less than 80 percent of patients in the treatment group were reported in a study, the data would not be used because of the high risk of bias.

### 2.9 Assessment of heterogeneity

Clinical heterogeneity will be assessed by comparing some significant participant factors, inclusion and exclusion criteria, interventions and outcomes, and trail factors, such as randomization concealment, blinding of outcome assessment, and losses to follow-up) among trails. Statistical heterogeneity will be evaluated by calculating I^2^^[19]^ or using Chi-Squared test. If the interventions are similar, we will evaluate heterogeneity by calculating I^2^, then analyze results in meta-analysis. If heterogeneity is high, we will use random-effects models.

If not, data will be analyzed using fixed effects model. When there is no statistical heterogeneity, we will use the fixed-effects model. Subgroups will be analyzed to assess the specific sources of heterogeneity if necessary.

### 2.10 Assessment of reporting biases

We will make a funnel plot to evaluate the possibility of report bias.

### 2.11 Data synthesis

We will use Rev Man version 5.0 to analyze. Results will be presented in the way the Cochrane Handbook recommended.^[17]^ For each trial, fixed-effect models and random-effect models will be used to compute the combined estimates. The fixed effects model will be used when the I^2^ statistic is less than 50%, for we assume the differences between each trial share equal chance, and if the I^2^ statistic is higher than 50%, the random-effects model will be used, for we assume each trial varies markedly. A systematic narrative synthesis will be conducted if it is impossible to complete any meta-analysis.

### 2.12 Subgroup analysis

If data permit, we will conduct subgroup analyses for:

(1) Different dosage and frequency of UDCA administration.
(2) Different removal procedure (laparoscopic surgery, Endoscopic Retrograde Cholangiopancreatography, Laparotomy, and so on) that patients have ever undergone.
(3) Different gallbladder state (underwent cholecystectomy or not, had gallstones or not) that patients had when they take UDCA as the prevention for the recurrence of CBDS.

### 2.13 Sensitivity analysis

If there are sufficient randomized trials are include, we will analyze sensitivity to evaluate the quality of the studies. We will remove 1 or several studies (high-risk bias) to explore the potential sources of heterogeneity and evaluate the impact of excluded trials on the total estimate through repeating the meta-analysis. In addition, we will assess the sample size and different models to insure the robustness of our results.

## 3 Ethics and dissemination

As this is a meta-analysis of published studies, there are no ethical or safety concerns. The results will be published in peer-reviewed journals. Our findings could provide a theoretical basis for UDCA to prevent recurrence of choledocholithiasis on accordance of the findings.

## 4 Discussion

UDCA is a potentially effective, having fewer side effects medicine for prevention of the recurrence after CBDS removal, though the large amount case-control study is limited and there is no systematic review before to evaluate the efficacy. Therefore, we conduct the review aiming to evaluate the effects of UDCA for prevention of recurrence after CBDS removal. Our findings will provide clinicians and health professionals a more leading-edge and objective evidence, and more and more CBDS patients may also benefit from these potential interventions.

## Author contributions

**Conceptualization:** Xun Chen, Xiao-Ru Yan, Li-Ping Zhang.

**Data curation:** Xun Chen, Xiao-Ru Yan.

**Formal analysis:** Xun Chen, Xiao-Ru Yan.

**Funding acquisition:** Li-Ping Zhang.

**Investigation:** Xun Chen, Xiao-Ru Yan.

**Methodology:** Xun Chen, Xiao-Ru Yan.

**Project administration:** Xun Chen, Xiao-Ru Yan.

**Resources:** Li-Ping Zhang.

**Software:** Xun Chen, Xiao-Ru Yan.

**Supervision:** Xun Chen, Xiao-Ru Yan, Li-Ping Zhang.

**Validation:** Xun Chen, Xiao-Ru Yan, Li-Ping Zhang.

**Visualization:** Xun Chen, Xiao-Ru Yan, Li-Ping Zhang.

**Writing – original draft:** Xun Chen.

**Writing – review & editing:** Xun Chen.
